# Screening Method to Evaluate Amino Acid-Decarboxylase Activity of Bacteria Present in Spanish Artisanal Ripened Cheeses

**DOI:** 10.3390/foods7110182

**Published:** 2018-11-06

**Authors:** Diana Espinosa-Pesqueira, Artur X. Roig-Sagués, M. Manuela Hernández-Herrero

**Affiliations:** CIRTTA—Departament de Ciència Animal i dels Aliments, Universitat Autònoma de Barcelona, Travessera dels Turons S/N, 08193 Barcelona, Spain; diespe@gmail.com (D.E.-P.); manuela.hernandez@gmail.com (M.M.H.-H.)

**Keywords:** biogenic amines, decarboxylase activity, screening method, artisanal cheese, high hydrostatic pressure

## Abstract

A qualitative microplate screening method, using both low nitrogen (LND) and low glucose (LGD) decarboxylase broths, was used to evaluate the biogenic amine (BA) forming capacity of bacteria present in two types of Spanish ripened cheeses, some of them treated by high hydrostatic pressure. BA formation in decarboxylase broths was later confirmed by High Performance Liquid Chromatography (HPLC). An optimal cut off between 10–25 mg/L with a sensitivity of 84% and a specificity of 92% was obtained when detecting putrescine (PU), tyramine (TY) and cadaverine (CA) formation capability, although these broths showed less capacity detecting histamine forming bacteria. TY forming bacteria were the most frequent among the isolated BA forming strains showing a strong production capability (exceeding 100 mg/L), followed by CA and PU formers. *Lactococcus*, *Lactobacillus*, *Enterococcus* and *Leuconostoc* groups were found as the main TY producers, and some strains were also able to produce diamines at a level above 100 mg/L, and probably ruled the BA formation during ripening. *Enterobacteriaceae* and *Staphylococcus* spp., as well as some *Bacillus* spp. were also identified among the BA forming bacteria isolated.

## 1. Introduction

Cheese is, after fish, the food product that most usually causes poisoning due to the presence of high amounts of biogenic amines (BA), compounds with psychoactive and vasoactive properties that can be formed in foodstuffs due to the microbial decarboxylation of amino acids [1,2,3,4,5,6]. Amino acid decarboxylase activity has been described for several groups of microorganisms, such as *Enterobacteriaceae*, *Pseudomonas* spp, *Enteroccoccus*, *Microccoccus* and Lactic Acid Bacteria (LAB). These BA-producing organisms may be part of the microbiota of the raw materials or may be introduced by contamination during or after processing of foodstuffs [4,7,8,9,10,11,12,13,14]. The specificity of the amino acid decarboxylases is strain dependent [11,15]. Lactic acid bacteria (LAB) have an important role in cheese elaboration and they are also the most important bacterial group that may build-up biogenic amine (BA), especially tyramine (TY) and putrescine (PU), but also cadaverine (CA) and histamine (HI) [13,16,17,18]. Sumner et al. [19] isolated a strain of *Lactobacillus buchneri* (strain St2A) from a Swiss cheese involved in an outbreak of HI poisoning occurred in the USA in 1980 that was able to form high amounts of HI. This LAB, later classified al *L. parabuchneri*, is able to grow and produce histamine at refrigeration temperatures [20]. *Enterobacteriaceae*, *Staphylococcus* spp. or *Bacillus* spp. have also been related to the accumulation of diamines in foods, including cheese, but also TY and/or HI [8,17,21,22].

Diverse qualitative and quantitative methods have been described in the literature to evaluate the amino acid decarboxylase activity of microorganisms isolated in food products. Different culture media have been proposed to be used as screening qualitative procedures, the most being formulated as a basal medium that include sources of carbon (glucose), nitrogen (peptone, yeast or meat extract), vitamins, salt, a relative high amount of one (or several) precursor amino acids and a pH indicator (e.g., bromocresol purple). Decarboxylase activity is then detected by the pH shift that changes the color of the medium when the carboxylic group is released from the amino acid(s) leaving in the medium the more alkaline BA(s) [23,24]. False-positive results have been described probably due to the formation of other alkaline compounds [9,22,25], but also false-negative responses are possible as a result of the fermentative activity of some bacteria, such as LAB, which produce acid that neutralize the alkalinity of BA [23,26].

In a previous work, the formation of BA in two artisanal varieties of Spanish ripened cheese, one made of ewe’s raw milk and other of goat’s raw milk, was presented. The effect of high hydrostatic pressure (HHP) treatments on both the levels of BA formed and on the main microbial groups present was also evaluated [27]. The aim of the present work has been to develop a fast, reliable and easy to perform screening method to evaluate the bacterial formation capacity of a wide range of BA, and evaluate the BA formation capability of the microbiota present in these two varieties of cheese to understand why HHP treatments reduce the formation of BA. 

## 2. Materials and Methods

### 2.1. Cheese Manufacturing

Two types of artisan ripened cheeses elaborated in Spain were studied in this survey, both made of enzymatic curd and pressed paste. The first one was produced from goat’s raw milk in the region of Catalonia, northeast of Spain, and the second was made from ewe’s raw milk in Castilla y León, central Spain. The procedure of sampling as well as the HHP treatment applied have been described in a previous work [27]. Three independent batches of each type of cheese were produced following the usual manufacturing procedures used by the manufacturers. Cheese samples were separated in three batches: samples not HHP treated (Control samples); samples HHP treated before the 5th day of ripening (HHP1) and samples treated after 15 days of ripening (HHP15, only for ewe’s milk cheeses). HHP treatments were performed at 400 MPa for 10 min at a temperature of 2 °C using an Alstom HHP equipment (Alstom, Nantes, France) with a 2 L pressure chamber.

### 2.2. Strain Isolation

Ten grams of each cheese sample were homogenized in 90 mL of sterile Buffered Peptone Water (Oxoid, Basingstoke, Hampshire, UK) with a BagMixer 400 paddle blender (Interscience, St Nom la Bretèche, France) and plated on M-17 agar (Oxoid) supplemented with a bacteriological grade lactose solution (5 g/L, Oxoid) and incubated at 30 °C, 48 h to isolate *Lactococcus* spp.; on de Man Rogosa Sharpe agar (MRS, Oxoid) incubated at 30 °C for 48 h to isolate Lactobacilli; on Kenner Fecal *Streptococcus* Agar (KF, Oxoid) supplemented with 2,3,5-triphenyltetrazolium chloride solution 1% (Oxoid) and incubated at 37 °C for 48 h to isolate Enterococci; Violet Red Bile Glucose Agar (VRBG, Oxoid) incubated at 37 °C for 24 h to isolate *Enterobactericeae* and Baird Parker Agar (BPA, BioMérieux, Marcy L’Etoile, France) incubated at 37 °C for 24–48 h to isolate *Staphylococcus* strains.

A total of 688 isolates were randomly picked out from the different selective media. The purification of each isolated was made by streaking single colonies on Petri plates with Tryptone Soy Agar (Oxoid) and incubating at 30 °C for 24–48 h. Two TY producing strains of *Lactobacillus brevis* and *Lactobacillus casei* and an HI producing strain of *Staphylococcus epidermidis*, isolated from previous surveys were used as positive controls [8,9]. These cultures were recovered in 10 mL of Tryptone Soy Broth (Oxoid) and incubated at 30 °C for 24 h. The purity of each culture was verified by subculturing the *Lactobacillus brevis* and *Lactobacillus casei* strains onto MRS agar (Oxoid), incubated at 30 °C for 24 h, and the *Staphylococcus epidermidis* strain on BPA (BioMérieux) incubated at 37 °C for 24 h. Before performing the decarboxylase assay, each strain was suspended in a tube with physiological solution of NaCl 0.85% (Panreac, Barcelona, Spain) until reaching a turbidity of about 0.5 in the McFarland scale.

### 2.3. Preparation of Decarboxylase Media

Table 1 shows the composition of the two synthetic media formulated to determine the ability to form the most toxic BA (HI and TY) and their enhancers (PU and CA): Low Nitrogen Broth (LND), prepared with the objective to decrease the incidence of false positive results of bacteria with a strong peptidase (or deaminase) activity; and the Low Glucose Broth (LGD) developed with the aim to decrease the incidence of false negative responses of bacteria with a great fermentative activity. Before performing the tests both base broth media were supplemented with the precursor amino acids (l-Lysine monohydrate (Merck, Darmstadt, Germany), l-Ornithine monohydrate (Sigma-Aldrich, Steinheim, Germany), l-Histidine monohydrochloride (Merck) and l-Tyrosine disodium salt (Sigma-Aldrich), individually, or adding a mixture of all them (described in the next section as total amino acid broth). The base broth without amino acids added was used as negative control. All media were adjusted to the pH values indicated in Table 1 and autoclaved at 120 °C during 5 min.

### 2.4. Assessment of Amino Acid Decarboxylase Activity

**Figure 1:**
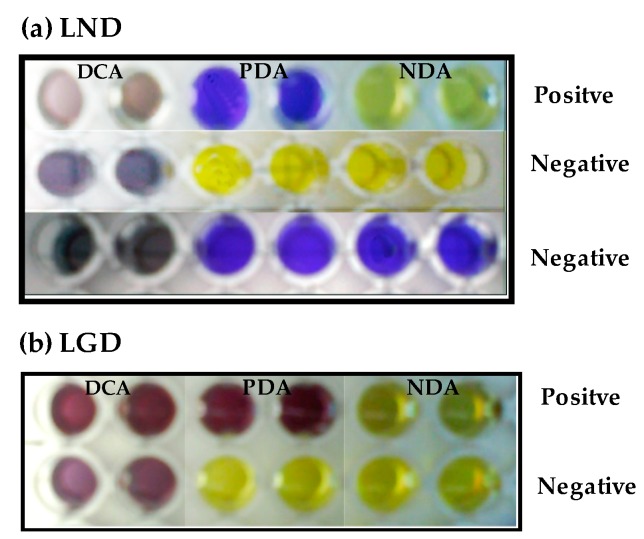
Example of negative and positive responses to amino acid decarboxylase activity in Low Nitrogen Decarboxylase (LND) and Low Glucose Decarboxylase (LGD) media. DCA: decarboxylase control assay; PDA: positive decarboxylase assay; NDA: negative decarboxylase assay.

In order to detect the capacity of the isolated strains to form BA and to determine which of the two decarboxylase broths (LND and LGD) show the best results in each one, a screening test was performed on a 96-wells flat bottom Microtiter plate. Aliquots of 200 µL of total amino acid broth (TAB) and 20 µL saline solution were added into 6 wells of a 96 well (decarboxylase control assay: DCA); 200 µL of TAB and 20 µL of bacterial suspension were added into another 6 wells (positive decarboxylase assay: PDA); and 200 µL of broth base without amino acids with 20 µL of bacterial suspension were added into another 6 wells (negative decarboxylase assay: NDA). Microplates were incubated at 30 °C for 24 h. A positive result was considered in PDA wells when a purple color appeared due to an increase of alkalinity (Figure 1a). In LGD broth positive results were also considered when no color changes were observed in PDA wells and yellow color appeared in NDA wells, because a high acidification was produced due to the bacterial growth (Figure 1b). Negative results were considered when no color changes were observed in PDA wells (Figure 1a,b), or when a purple color appeared in NDA wells due to another alkaline compounds different than BA (Figure 1a).

### 2.5. Confirmation of Amino Acid Decarboxylase Activity by HPLC

Decarboxylase activity of strains was confirmed by the quantitative analysis of BA produced in the decarboxylase broths by means of reverse-phase High Performance Liquid Chromatography (HPLC), using an automated HPLC system (HPLC P680, Dionex, Sunnyvale, CA, USA) equipped with an Ultra Violet (UV) detector Dionex UVD170U (Thermo-Fisher Scientific, Waltham, MA, USA). Briefly: one mL of each bacterial suspension (0.5 McFarland) was inoculated into a tube containing 4 mL of the TAB version of LND or LGD broths (depending on the previous results for each strain). After 4 days of incubation at 30 °C, the media was centrifuged (9000× *g*, 10 min, 20 °C) and 3 mL of the supernatant was extracted with 2 mL of 0.4 M HClO_4_ (Panreac). Determination of BA was carried out according to the RP-HPLC method described by Eerola et al. [28] and modified by Roig Sagués et al. [8] using dansyl chloride reagent (Sigma-Aldrich Chemical) to derivate the sample. The separation was performed on a Waters Spherisorb S5 ODS 2 45 × 150 mm column (Waters Corporation, Milford, MA, USA). All reagents were of analytical grade and all solvents involved in derivatization and in the separation process were of HPLC grade. The BA standards: putrescine (PU), cadaverine (CA), histamine (HI), tyrosine (TY), and the internal standard 1,7-diaminoheptane, were all purchased from Sigma-Aldrich Chemical.

### 2.6. Analytical Validation of the Qualitative Microplate Method of Amino Acid Decarboxylase Activity

The sensitivity, specificity, and the positive and negative predictive values were obtained to determine the diagnostic properties of the qualitative method [29,30,31] and were calculated by the following equations:

$$\;\mathrm{Sensitivity}=\frac{\mathrm{TP}}{\mathrm{TP}+\mathrm{FN}}\;\times 100\;$$

where TP is the truly positive amino acid decarboxylating isolates, correctly identified by the screening test and FN is the false negative responses obtained.

$$\;\mathrm{Specifity}=\frac{\mathrm{TN}}{\mathrm{TN}+\mathrm{FP}}\;\times 100\;$$

where TN is the truly negative (TN) amino acid decarboxylating isolates, correctly identified by the screening test and FP is the false positive responses obtained.

$$\;\mathrm{PPV}=\frac{\mathrm{TP}}{\mathrm{TP}+\mathrm{FP}}\times 100\;$$

where PPV is the positive predictive value and reflects the proportion of truly positive isolates confirmed by HPLC among all positive isolates evaluated by Microtiter plate screening.

$$\;\mathrm{NPV}=\frac{\mathrm{TN}}{\mathrm{TN}+\mathrm{FN}}\times 100\;$$

where negative predictive value (NPV) is reflects the proportion of truly negative isolates confirmed by HPLC among all negative isolates evaluated by Microtiter plate screening.

The Receiver Operating Characteristic (ROC) curves were assessed using the MedCalc statistical software, version 11.2.1 (MedCalc, Ostend, Belgium), to know the discriminative power of the qualitative method referred to the HPLC method with its 95% confidence interval. In a ROC curve the true positive rate (Sensitivity) is plotted in function of the false positive rate (100-Specificity). A test with perfect discrimination (no overlap in the two distributions) has a ROC curve that passes through the upper left corner (100% sensitivity, 100% specificity). Therefore, the closer the ROC curve is to the upper left corner, the higher the overall accuracy of the test [32]. The area under the ROC curve (AUC) is a measure of how well a parameter can distinguish between two groups (isolates with amino acid decarboxylase activity/isolates without this capacity). The better overall diagnostic performance of the test is when the AUC value is closer to 1 and the practical lower limit for the AUC of a diagnostic test is 0.5 [31,33]. A classification of diagnostic accuracy for the qualitative method is given according to AUC value: AUC 0.90–1.0 excellent, 0.80–0.90 good, 0.70–0.80 fair, 0.60–0.70 poor, 0.50–0.60 deficient and 0.50 null [34]. 

The point of intersection of the ROC curve with the diagonal line drawn from 100% sensibility to 100% 1-specificity was chosen as the best discriminator value. The optimal cut-off value showed the highest accuracy, the lowest false negative (FN) and the highest false positive (FP) results.

### 2.7. Identification of Strains with Decarboxylase Activity

Confirmed decarboxylase-positive strains were identified based on Gram stain and catalase and citochromooxidase activity [35]. Further identification to the species level was carried out by a variety of biochemical tests using API 20-E, API 20-Strep, API-Staph and API 50-CH strips (BioMérieux, Marcy l’Etoile, France). 

## 3. Results and Discussion

### 3.1. Validation of the Qualitative Microplate Method of Amino Acid Decarboxylase Activity

ROC curve analysis was used to determine the discriminative power and the cuts-off of the amino acid decarboxylase screening method with both media (LND and LGD) to evaluate the specific amino acid decarboxylase activity (Table 2).

Tyrosine decarboxylase test showed an area under the ROC curve (AUC) around 0.98, with an optimal cut-off value at 25 mg/L and 20 mg/L of TY on LND and LGD broths, respectively. This means that the microplate screening method could discriminate the isolates with tyrosine decarboxylase activity the 98% of the time at optimal cut-off. The sensitivity and specificity values obtained with both broths were higher than 92%, reflecting that the number of false negative (FN) and false positive (FP) responses obtained by the qualitative method were generally low. However, the negative predictive value (NPV) was considered low (<66%). AUC for the lysine decarboxylase test displayed was greater than 0.930 with an optimal cut-off concentration of 15 mg/L and 10 mg/L for LND and LGD broths, respectively. In this case, the sensitivity and specificity values using LND broth were about 98% and 93%, respectively, while for LGD broth values were about 88% and 98%, respectively. The assay to detect ornithine decarboxylase with the LND broth showed the highest diagnostic values (over 98%) with the lowest cut-off concentration (10 mg/L) and an AUC higher than 0.995. On the other hand, for the same test using LGD broth an 84% of sensitivity and a 97.5% of specificity were reached at a cut-off value of 15 mg/L with an AUC of 0.907. 

Histidine decarboxylase test showed the lowest sensitivity values (below 60%) using both broths with the highest optimal cut-off concentration set at 50 mg/L with specificity values up to 90%. Likewise, the AUC value was the lowest, possibly due to a 16.5% of FP and 24% of FN reactions observed at the optimal cut-off using LND broth, whereas a 9.9% and 25% of FP and FN were obtained, respectively, in LGD broth.

In many occasions it has been reported that qualitative screening decarboxylase methods have some limitations in terms of sensitivity in detecting BA production. The presence of FP and FN reactions reported has not been insignificant. Hernández-Herrero et al. [9] observed that 96.5% of the suspected histamine formers detected by Niven decarboxylase media were finally considered as FP. Likewise, Roig-Sagués et al. [36] found that only a 15.8% of the total presumptively histamine-formers obtained in Joosten and Northolt media [37] were confirmed. Similar results were observed when tyramine decarboxylase capacity was tested in the same media, where only 8.4% of the suspected isolates with tyrosine decarboxylase activity were confirmed. The FP results were attributed to the production of other substances able to alkalinize the media [25]. Similarly, Moreno-Arribas et al. [38] used the Maijala modified decarboxylase media and noticed a high number of FP reactions to PU and agmatine production, but less than were found in the tyrosine decarboxylase activity test. On the contrary, de las Rivas et al. [15] did not find any correlation between the positive responses in the decarboxylase activity media and the BA detected by HPLC. They suggested that the screening Maijala modified decarboxylase media underestimates the number of BA-producing strains. On the contrary, Bover-Cid and Holzapfel [23], in their improved screening media tested on LAB, did not observe FP reactions and only 3 strains showed a negative response with the screening procedure. They justified these FN results due to the low amount of tyramine formed that did not neutralize the acid production of LAB. Although these authors proposed their improved decarboxylase medium as a rapid preliminary method to select strains with low decarboxylase activity, the optimal cut-off value was around 300 mg/L. Torracca et al. [17] reported, using the same decarboxylase medium described by Bover-Cid and Holzapfel [23], an optimal cut-off value of 631 mg/L and 810 mg/L for PUT and TY, respectively.

### 3.2. Amino Acid Decarboxylase Activity of the Control Strains

Table 3 shows the results after testing the control strains in the microplate screening method and the result of the confirmation by HPLC. Lactobacillus brevis and Lactobacillus casei showed tyrosine decarboxylase activity in LGD broth, and Staphylococcus epidermidis histidine decarboxylase capacity in LND broth. All strains were also able to produce low amounts of PU (around 1 mg/L) but were only detected by HPLC.

### 3.3. Biogenic Amine Production by Isolates from Goat’s and Ewe’s Milk Cheeses

#### 3.3.1. Total Amino-Acid Decarboxylase Activity of the Isolated Strains 

A total of 688 strains were obtained from the different culture media and a 43.02% of them gave a positive response in the microplate assay with TAB, being subsequently confirmed by HPLC. A 37.7% of the bacteria isolated from goat’s milk cheeses and a 47% of the strains picked up from ewe’s milk cheeses were BA-formers. The number of isolates obtained from VRBG and BPA media was much lower since these two groups of microorganisms are a minority among the microbiota of ripened cheeses, and their counts are usually low [27], but the percentage of decarboxylase positive results among these isolates was higher (87.5% and 92% on VRBG and BPA, respectively) than in KF, M-17 and MRS media (49.7, 36.8, and 35% respectively). Several studies found that the decarboxylase activity is more frequent in Enterobacteriaceae strains (from 80 to 95%) and in less extension among LAB strains (from 9.5 to 65%) [8,21,36,38,39]. Nevertheless, Enterobacteriaceae and Staphylococcus usually do not achieve high counts in ripened cheeses made under good hygienic practices and normally become undetectable after few days of ripening, reason why decarboxylase positive LAB is usually considered the main responsible for the formation of high concentrations of BA in cured cheeses [17,21].

#### 3.3.2. Specific Amino Acid Decarboxylase Activity of the Isolated Strains

The assessment of the specific amino acid decarboxylase activity was done with the strains that gave positive responses in the screening assay with TAB. Up to 150 of these isolates were recovered and tested in LND and LGD media, respectively. In general, the capability to decarboxylate tyrosine was the most frequent activity detected (91.6% of the total isolates tested) in the specific amino acid decarboxylase screening assay, followed by the ability to decarboxylate lysine and ornithine (around 33.5%). In these cases, the 95%, 96% and 94% were confirmed by the HPLC analysis, respectively. However, histidine decarboxylase activity was detected in only a 24% of the isolates tested, a 76% of them confirmed by HPLC.

Table 4 shows the number of strains with HPLC-confirmed BA-producing capability obtained from goat´s and ewe’s milk cheeses according to the culture media of origin. These results are shown as a whole without considering the HHP treatment to which cheese samples were subjected. Strains were grouped in four categories according to Aymerich et al. [38]: medium amine formers (25–50 mg/L), good amine formers (50–100 mg/L), strong amine formers (100–1000 mg/L) and prolific amine formers (>1000 mg/L). In general, strong amine formers were more frequent among strains with TY-producing capability, followed by those able to form CA and PU. Prolific amine production capacity was only observed in some diamine formers, while the formation of HI in amounts above 100 mg/L was a rare event (Table 4).

The isolates obtained from VRBG medium showed the highest frequency of PU and CA forming activity. Lysine and ornithine decarboxylases are very common among enterobacteria, and their detection is commonly used for the biochemical identification of *Enterobacteriaceae* species. A 100% of the isolates picked up from goat’s and ewe’s milk cheeses showed strong activity (>100 mg/L) for CA, and 87.5% and 85.35% for PU, respectively. Strong TY forming capacity was detected in only one isolate obtained from goat’s milk cheeses and in 8 from ewe’s milk cheese. The isolates with histidine decarboxylase activity showed a weak production and only one isolate obtained from goat’s milk cheese and three from ewe’s milk cheese presented the ability to produce more than 100 mg/L. *Enterobacteriaceae* are known to decarboxylate several amino acids, specially arginine, lysine and ornithine [11,39,40,41] and histidine [17,25,42]. The number of isolates with amino acid decarboxylase activity obtained from BPA culture medium was low. Between 75–100% of these isolates displayed a strong PU, CA and TY forming ability. Whereas histamine accumulation was detected especially in a range of 25–100 mg/L in the 89% of the cases. Little information is available about the production of BA by *Staphylococcus* spp. in cheese. However, some species of this group have been related to a variable formation of TY, PU, CA and/or HI in meat and fish fermented products. Martin et al. [43] found in fermented sausages that TY was the main amine produced by this group and some strains also were able to produce PU, CA and HI. Hernández-Herrero et al. [9] reported that the main HI-formers detected in salted anchovies belonged to this genus and de las Rivas et al. [15] reported some strains isolated from “Chorizo”, a Spanish ripened sausage, as TY-formers. Most of the isolates obtained from the KF medium were strong TY formers (76% from goat’s milk cheese and 81% from ewe’s milk cheese) and some of them were also able to produce above 100 mg/L of PU and CA. The ability to form TY was also frequent in the strains isolated from M-17 medium in both kind of cheeses (79% from goat’s and 65% from ewe’s milk cheeses, respectively) in amounts above 100 mg/L. In that case, the ability to form diamines was less frequent (around 5% of the isolates) in both type of cheeses. Similar results were found in the isolates obtained from MRS medium where around 69% of them were considered strong TY-producers, but no PU or CA formation was detected in any of the isolates obtained from goat’s milk cheese and only a 10% of those obtained from ewe’s milk cheese were able to from up to 100 mg/L.

#### 3.3.3. Identification of BA-Producing Strains

The result of the identifications of the BA-forming strains isolated from cheese samples, as well as their BA forming capacity expressed as mg/L, are shown in Table 5 (goat’s milk cheese) and Table 6 (ewe’s milk cheese). It was not possible to establish a clear effect of the HHP treatments to which some of the cheese samples were submitted on the type of strain and its amine-forming capacity. Consequently, results are shown globally, without considering the type of treatment to which the samples were submitted. In both types of cheese, most of the strains with decarboxylase activity were Gram positive. In the case of the goat’s milk cheese only 5 Gram negative strains showed decarboxylase activity, four of them identified as *Hafnia alvei*, all of them with a strong PU and CA forming capacity. One of these strains also showed a strong capacity to form TY, but much lower than diamines. Nevertheless, the maximum capacity to form these amines (and also HI) was shown by a strain that could not be precisely identified. In the case of the ewe’s milk cheese, 11 Gram negative strains showed decarboxylase activity, most of them (5) also identified as Hafnia alvei and one of them showing the maximum capability to form CA, PU and HI. The maximum TY-forming capacity was shown by a strain of *Citrobacter freundii*. Strains of *Klebsiella oxytoca* and *Escherichia coli* with strong diamine production capacity were identified from ewe’s cheese samples. However only one strain of each specie and other identified as *C. freundii* were able to produce CA and TY in a considerable amount (above 100 mg/L). *H. alvei, K. oxytoca* and *E. coli* have been previously associated with the formation of PU, CA and/or HI in foods [8,9,11,17,21,25,36,41]. Also, some strains of these species have been reported to possess the ability to decarboxylate tyrosine [21]. The formation of high amounts of PU and CA, as well as of TY, has been previously reported by *C. freundii* [21,40], indicating that this specie is more prolific forming PU than CA. Enterobacteria is usually a minor group among the microbiota present on fermented products. In the cheese object of this work, enterobacteria counts were usually below 3 Log_10_ after 60 days of ripening, but their counts at the beginning of the process were above 6 Log_10_ [27]. When unhygienic manufacturing practices allow for achieving high counts of enterobacteria at the beginning of the process, the fact that their counts would be later reduced during ripening does not necessarily imply the inhibition of their decarboxylase activity and consequently may contribute to BA formed in the final product [36].

Among strains identified as Gram positive, 50 that showed decarboxylase activity were obtained from the goat’s milk cheese and 55 from the ewe’s milk cheese, and in general this activity was much higher than the Gram negative strains. Among the positive decarboxylase bacteria isolated from the ewe’s milk cheeses one strain of *Staphylococcus chromogenes* showed to be a prolific diamine former and strong TY former. Likewise, strains of *Staphylococcus xylosus* and *Staphylococcus aureus* with strong TY and HI production, respectively, were also found. On the other hand, the most frequent strains with high BA forming capacity obtained from goat’s milk cheeses were identified as *Staphylococcus hominis*, that were capable to produce high levels of PU, CA and TY, and *Staphylococcus warneri*, which showed to be a strong diamine producer.

*S. chromogenes* was previously reported as a prolific PU former, good CA and strong TY and HI-forming bacteria in Spanish salted anchovies [44]. Masson et al. [45] detected a weak TY-production capacity in strains of *S. xylosus* isolated from fermented sausages and Martin et al. [43] found in slightly fermented sausages some strains of *S. xylosus* capable to produce strong and prolific amounts of TY, PU and/or HI, and in a lesser extent of CA. Silla-Santos [46] observed HI-production in the 76% of *S. xylosus* strains isolated from Spanish sausages. Strains of *S. warneri* have also been reported to possess tyrosine decarboxylase, but with great variability of production [15,43,45]. CA and PU formation, in medium-good and strong concentration, have also been described [39]. Drosinos et al. [47] isolated one strain of *S. hominis novobiosepticus* in traditional fermented sausages with lysine and tyrosine decarboxylase activity. As enterobacteria, *Staphylococcus* spp. are associated to the contamination of food during unhygienic handling, and consequently it is important to follow always good manufacturing practices to avoid the proliferation of this kind of bacteria in the product. Nevertheless, in the cheeses from where the studied strains were obtained *Staphylococcus* spp. counts were always low (below 3 Log_10_ at the beginning of the ripening), and their counts reduced during ripening until being undetectable after 60 days in most cases.

*Enterococcus faecalis*, *Enterococcus faecium* and *Enterococcus durans* were the most frequent amine producing bacteria identified from both types of cheese, with a varied production capacity of TY, PU and CA (Table 5 and Table 6). In addition, one strain of *E. faecium* and two of *E. durans* showed a strong HI formation capacity. Enteroccoci are commonly associated with unhygienic conditions during the production and processing of dairy, although they can play an important role for developing the aroma and flavor of certain type cheeses, especially traditional cheeses produced in the Mediterranean area [48]. Several authors have described *E. faecalis, E. faecium* and *E. durans* as the most frequently TY formers in food [12,17,21,23,25,49,50,51,52] and also some strains of *E. faecalis* and *E. faecium* have been registered as capable of producing amounts up to 100 mg/L of PU [12,17,21,53] and CA [17,21,50] and/or HI [17,21,54]. Tyrosine is a relevant amino acid for the formation of BA in cheese as it can be an inducer of PU production in *E. faecalis* and would be received by the enterococci cells as a signal to growth, what would lead in an increment in the number of BA-producing cells increasing the risk of accumulating TY and PU in cheese [55]. No references concerning histidine decarboxylase activity of *E. durans* have been found in the literature.

Several BA producing strains isolated from M-17 and MRS media of goat’s milk cheeses were identified as *Lactococcus lactis* subsp. *lactis*, followed by Lactobacillus brevis, *Lactobacillus plantarum*, and *Leuconostoc* spp. All of them showed strong TY forming ability and *Leuconostoc* spp. also showed a strong-prolific PU formation. In the case of ewe’s milk cheeses, *L. lactis* subsp. *lactis*, *L*. *lactis* subsp. *cremoris*, *Pediococcus pentosaceus*, *Lactobacillus paracasei* subsp. *paracasei*, *L. plantarum* and *Leuconostoc* spp. were often associated with a strong TY-forming capability. Moreover, two strains identified as *L. lactis* subsp. *lactis* showed strong PU and CA forming ability, respectively, while two strains of *P. pentosaseus* species were strong PU and prolific CA producers, respectively. 

Several studies have reported different species of LAB able to form BA, especially TY [8,11,16,17,18,21,23,25,50,54,55,56,57], but also HI [58,59]. Within the species of LAB that may occur in food some strains of *L. brevis* and *L. plantarum* were reported to possess the potential to form TY, PU and/or HI [15,18,21,23,25,60,61]. Strains of *P. pentosaceus* isolated from commercial starters [26] and ripened sausages [25] were reported to form TY. Although *L. lactis* subsp. *lactis*, *L. lactis* subsp. *cremoris* and *L. paracasei* subsp. *paracasei* are species usually used as starter cultures or probiotic strains and usually reported as non-decarboxylating strains [16,17,18,62], some strains of *L. lactis* and *L. paracasei* have been reported with the ability to form TY, HI, PU and/or CA in amounts up to 1000 mg/L [8,17,18,21,23,25,51,58] and *L. lactis* subsp. *cremoris* has been also described as a PU-producer [12]. *Leuconostoc* spp. has been frequently described among the microbiota present in several Spanish farm house cheeses [3,63,64], and has also been described to possess tyrosine, lysine and/or histidine decarboxylase activity [39]. Likewise, González de Llano et al. [63] reported the production of TY in a range of 100–1000 mg/L by strains of *Leuconostoc*. Pircher et al. [21] found that strains of this genus could form TY, PU, CA and /or HI in amounts up to 100 mg/L. 

Strains belonging to the *Bacillus* genus isolated from ripened salted anchovies, cheese and raw sausages have previously been described as BA-formers [9,22,36]. One of them was *Bacillus macerans* isolated from Italian cheese, which was capable to from prolific amounts of HI. Formation of CA and PU was also observed in this strain [22].

### 3.4. Consequences of HHP Treatments on the Formation of BA

The effect of the HHP treatments on the microbial counts, the proteolytic activity and the formation of BA in the cheeses from where we obtained the studied strains are described in the previous work of Espinosa-Pesqueira et al. [27]. TY and PU were the main BA formed in control (untreated) cheeses, whereas in ewe’s milk chesses the level of CA was also relevant. However, in cheeses that were pressurized on the 5th day of ripening (HHP1) the amounts TY formed at the end of the ripening (60th day) were about 93% and 88% lower than in control goat’s and ewe’s raw milk cheeses, respectively, and similar was the result for PU. The application of an HHP treatment on the 15th day of ripening (HHP15) showed to be less efficient reducing the formation of BA. HHP1 treatments caused a significant decrease on microbiological counts, specially of LAB, enteroccocci and enterobacteria, and also reduced the proteolytic activity, showing a reduction of about 34% and 49% of the free amino-acid content in goat’s and ewe’s cheeses, respectively. Although HHP15 samples also reduced the microbial counts, this did not affect the proteolysis, and consequently the release of amino acids. Several authors mentioned that the specificity of the amino acid decarboxylases is specially strain dependent [11,15,39,60] and a great variability in BA production by different groups and species of bacteria, either in type or amount, was found in this survey. LAB and enterococci were among the most efficient TY producers found in this work. LAB ruled the ripening process of cheeses from the beginning, especially *Lactococcus*, becoming the most important group of microorganisms in the ripened cheese. 

The application of the HHP treatments in the early stages of ripening (HHP1) caused a significant reduction of LAB counts, although they recovered along the ripening process. It should be considered that the BA forming rate is greater during the first 15–30 days of ripening, and consequently, the reduction on the counts of the most prolific BA forming groups at these stages could have contributed to reduce the decarboxylase potential, as well as the availability of amino acids, reducing the final amounts of BA. Enterococci were also affected by HHP1 treatments and could not recover their initial counts during the rest of the ripening, affecting their contribution to the formation of BA. 

The role of other minority bacterial groups, such as enterobacteria, on the BA formation is not so clear. In the cheeses object of this work their counts were above 6 Log_10_ at the beginning of the ripening, although practically disappeared after both HHP1 and HHP15 treatments samples. Different enterobacteria have been identified as prolific PU, CA and even TY formers, and consequently its elimination the first days of ripening could have reduced their contribution to the amino acid decarboxylase activity. When treatments are applied after 15 days of maturation, the decarboxylase capacity of these microorganisms in the early stages of maturation was still present and consequently their elimination from the 15th day of ripening reduced the consequences on the BA formation.

## 4. Conclusions

The microplate screening method allows for a rapid preliminary selection of strains with low decarboxylase activity, with a detection limit estimated around 50 mg/L. Moreover, the use of Microtiter plates allows for processing a large numbers of samples, reducing the volume of material and culture media needed. The data indicates that, in general, the specific amino acid decarboxylase assay with LND and LGD broths have satisfactory diagnostic parameters to discriminate bacterial isolates with ornithine, lysine and tyrosine decarboxylases. Moreover, the sensitivity and specificity values for ornithine, lysine and tyrosine decarboxylase test with both types of media were acceptable with low numbers of FP and FN responses. Generally, FN responses were due to weak BA producers. The detection of histidine decarboxylase activity in bacterial isolates using LND and LGD broths have a low sensitivity, but a high specificity value. About a 43% of the strains isolated from cheeses showed decarboxylase activity on one or more amino acids and most of them were later confirmed, especially of TY, PU and CA that were the most important present in cheeses after ripening. The application of the HHP treatments, especially in the early stages of ripening, caused a significant reduction among the most prolific BA forming groups including LAB. Considering that the BA forming rate is greater during the first 15–30 days of ripening, this effect on the microbiota reduces the decarboxylase potential, as well as the availability of amino acids, reducing the final amounts of BA formed. Most of the strains were LAB, including some species that are important for the development of the typical cheese characteristics, such as *L. lactis*, that can be used as starter culture, or *Lactobacillus casei/paracasei*, *Lactobacillus plantarum* and *Lactobacillus curvatus*, of which there is increasing interest to be employed in dairy products with ‘protected geographic indication’ [18]. Consequently, the formation of BA should always be considered among the selecting criteria for strains considered as suitable to be used as starter cultures. 

## Figures and Tables

**Table 1:** Composition of broth media (g/100 mL)) used to evaluate decarboxylase ability of strains isolated from cheeses.

Reagent	Low Nitrogen Decarboxylase Broth (LND)	Low Glucose Decarboxylase Broth (LGD)	Adjusted pH
Tryptone	0.125	0.25	
Yeast extract	0.125	0.25	
NaCl	0.25	0.25	
CaCO_3_	0.01	0.01	
Pyridoxal-5-phosphate	0.03	0.03	
Glucose	0.05	0.001	
Bromocresol purple	0.01	0.01	
All amino acids	1.0	1.0	5.5
l-Lysine	1.0	1.0	5.0
l-Ornithine	1.0	1.0	5.5
l-Histidine	1.0	1.0	5.7
l-Tyrosine	0.25	0.25	5.5

**Table 2:** Receiver Operating Characteristic (ROC) curve analysis of qualitative method to predict specific amino acid decarboxylase activity in isolates using Low Nitrogen Decarboxylase (LND) and Low Glucose Decarboxylase (LGD) broths.

Broth	Data of ROC Analysis	Ornithine	Lysine	Histidine	Tyrosine
**LND**	AUC	0.999 (0.974–1.00)	0.992 (0.961–1.00)	0.737 (0.659–0.806)	0.980 (0.943–0.996)
	Optimal cut-off (mg/L)	10	15	50	25
	Sensitivity at optimal cut-off (%)	98.53	98.68	65.38	92.25
	Specificity at optimal cut-off (%)	100	93.15	91.75	100
	PPV (%)	100	83.7	81	100
	NPV (%)	99.0	98.6	83.2	66.7
	FN at cut-off	3	6	11	1
	FP at cut-off	1	1	17	6
**LGD**	AUC	0.907 (0.849–0.948)	0.935 (0.883–0.969)	0.592 (0.509–0.672)	0.989 (0.956–0.999)
	Optimal cut-off (mg/L)	15	10	50	20
	Sensitivity at optimal cut-off (%)	84.37	88	30	93.06
	Specificity at optimal cut-off (%)	97.5	97.6	100	100
	PPV (%)	90	88	100	100
	NPV (%)	95.8	97.6	90.3	37.5
	FN at cut-off	5	5	2	0
	FP at cut-off	5	3	14	8

AUC: Area under ROC curve and 95% confidence interval; PPV: Positive predictive value; NPV: Negative predictive value; FN: Number of false negative: FP: Number of false positive.

**Table 3:** Biogenic amine production by positive control bacteria strains in the amino acid decarboxylase microplate assay (DMA) and HPLC analysis (mg/L).

Strain		PU		CA		HI		TY	
	Broth	DMA	HPLC	DMA	HPLC	DMA	HPLC	DMA	HPLC
*L. brevis*	LGD	(−)	1.11	(−)	ND	ND	ND	(+)	109.8
*L. casei*	LGD	(−)	0.78	(−)	ND	ND	ND	(+)	77.14
*S. epidermidis*	LND	(−)	0.85	(−)	ND	(+)	46.48	(−)	ND

PU: putrescine; CA: cadaverine; HI: histamine; TY: tyramine; LND: Low Nitrogen Decarboxylase; LGD: Low Glucose Decarboxylase; DMA (detection on microplate assay): (+) Positive; (−) Negative; ND: not detected.

**Table 4:** Biogenic amine forming capacity of bacteria isolated from goat´s and ewe’s milk cheeses according to the culture media. High hydrostatic pressure (HHP) treatments to which cheese samples were subjected are not considered.

Medium	BAP	PU				CA				HI				TY			
		±	+	++	+++	±	+	++	+++	±	+	++	+++	±	+	++	+++
VRBG	25	1	2	17	4	0	0	17	7	8	10	4	0	3	6	9	0
BPA	15	1	1	3	3	0	0	4	3	4	4	1	0	2	2	7	0
KF	89	5	3	22	4	13	0	22	9	19	21	4	0	4	8	71	0
M-17	98	7	2	5	0	7	1	2	1	14	6	2	0	5	6	69	0
MRS	72	2	2	2	3	0	0	2	3	7	0	0	0	5	8	50	0
Total	299	16	10	49	14	20	1	47	23	52	41	11	0	19	30	206	0

BAP: Number of positive BA producers; PU: putrescine; CA: cadaverine; Hi: histamine; TY: tyramine; Number of BA-forming isolates detected depending on their production (in mg L^−1^): (±) 25–50, medium; (+) 50–100, good; (++) 100–1000, strong; and (+++), >1000, prolific.

**Table 5:** Identification of strains obtained from goat’s raw milk cheeses with decarboxylase activity and their BA production in decarboxylase broths (mg/mL). Results are shown without considering the HHP treatments to which some cheese samples were subjected.

Identification	*N*	PU		CA		HI		TY	
**Gram Negative**									
*Hafnia alvei*	4	4	537.7–889.54	2	641–1001.30	4	30.43–95.68	4	22.44–151.54
*Enterobacteriaceae*	1	1	1037.52	1	1173.24	1	111.43	1	73.41
**Gram Positive**									
*Staphylococcus cohni* subsp. *cohni*	1	-		1	1.50	1	1.57	1	25.78
*Staphylococcus warneri*	2	2	69.29–753.55	2	240.94–694.22	1	88.63	1	65.50
*Staphylococcus capitis*	1	1	310.30	1	246.37	1	95.13	-	
*Staphylococcus lentus*	2	1	23.44	2	5.41–19	-		2	39.58–191.69
*Staphylococcus hominis*	2	1	890.96	1	998.20	1	91.63	2	102.42–245.22
*Enterococcus faecalis*	8	8	39.66–884.27	8	32.44–972	6	25.62–92.35	8	327.5–477.20
*Enterococcus durans*	2	-		-		-		2	337.40–357.44
*Enterococcus avium*	1	-		1	26.52	1	56.51	1	24.88
*Enterococcus faecium*	6	3	19.68–1113.80	3	1.32–1281.50	3	20.22–111.38	6	9.91–366.47
*Lactococcus lactis* subsp. *lactis*	10	1	7.25	1	7.50	2	21.24–33.38	10	198.77–450.77
*Pediococcus pentosaceus*	1	-		-		1	33.44	1	55.71
*Lactobacillus brevis*	5	-		-		1	22.51	5	212.58–519.52
*Lactobacillus plantarum*	3	1	22.41	-		1	24.5	3	307.62–528.45
*Lactobacillus paracasei* subps. *paracasei*	1	-		-		-		1	307.03
*Leuconostoc* spp.	3	2	252.88–749.05	2	14.47–28.83	2	22.74–25.75	3	330.01–417
*Bacillus macerans*	1	-		-		-		1	418.10
*Bacillus licheniformis*	1	-		-		-		1	403.07

N: number of strains identified; PU: putrescine; CA: cadaverine; HI: histamine; TY: tyramine.

**Table 6:** Identification of strains obtained from ewe’s raw milk cheeses with decarboxylase activity and their BA production in decarboxylase broths (mg/mL). Results are shown without considering the HHP treatments to which some cheese samples were subjected.

Identification	*N*	PU		CA		HI			TY
**Gram Negative**									
*Escherichia coli*	2	2	746–857.48	2	762.35–983.93	2	41.37–74.12	2	21.7–281.48
*Hafnia alvei*	5	5	738.50–1049.51	5	787.20–1180.12	4	43.52–185.54	5	48.83–184.79
*Klebsiella oxytoca*	2	2	30.63–458.50	2	493.41–866.96	2	16.04–27.22	2	5.01–167.24
*Citrobacter freundii*	1	1	67.1	1	1095.3	1	45.62	1	372.38
*Enterobacteriaceae*	1	1	832.76	1	846.95	1	83.95	1	69.42
**Gram Positive**									
*Staphylococcus xylosus*	2	-		-		2	42.31–68.5	2	92.09–475.35
*Staphylococcus chromogenes*	1	1	1142.92	1	1760.06	1	31.12	1	441.40
*Staphylococcus aureus*	1	-		-		1	100.54	-	
*Enterococcus faecalis*	5	3	860.29–978.9	4	35.43–1394.87	5	20.7–92.98	5	338.01–461.50
*Enterococcus durans*	9	9	12.63–1160.44	8	13.32–1773.03	9	30.8–179.32	9	46.09–747.33
*Enterococcus faecium*	2	2	24.89–847.05	2	877.24–941.69	2	28.98–29.97	2	100.54–149.41
*Enterococcus hirae*	2	2	552.4–579.25	2	600.67–615.75	2	32.36–45.95	2	108.01–187.58
*Enterococcus avium*	2	-		1	15.17	1	2.18	2	19.93–434.25
*Streptococcus salivarius*	1	-		-		-		1	742.1
*Lactococcus lactis* subsp. *lactis*	9	3	34.39–795.26	2	14.95–844.13	2	16.38–30.72	9	211.93–566.2
*Lactococcus lactis* subsp. *cremoris*	4	3	4.78–37.63	3	5.23–22.94	2	11.15–19.03	4	229.47–406.87
*Pediococcus pentosaceus*	4	3	9.91–897.67	3	10.25–1018.4	3	27.06–88.78	4	34.58–411.66
*Lactobacillus paracasei* subsp. *paracasei*	6	2	17.42–45.05	1	17.18	1	51.93	6	365.79–575.73
*Lactobacillus plantarum*	4	1	8.58	1	17.58	1	26.26	4	45.1–353.31
*Lactobacillus brevis*	1	-		-		-		1	240.08
*Lactobacillus pentosus*	1	1	50.79	-		-		1	422.75
*Leuconostoc* spp.	5	2	11.13–1162.76	2	22.52–1781.26	3	29.63–33.82	5	392.01–626.42

*N*: number of strains identified; PU: putrescine; CA: cadaverine; HI: histamine; TY: tyramine.
